# Supplementary material for: Relationship between Dietary Fiber Intake and the Prognosis of Amytrophic Lateral Sclerosis in Korea
Source: Nutrients. 2020 Nov 7;12(11):3420. doi: 10.3390/nu12113420 (PMC7695159; doi:10.3390/nu12113420)
Supplement: Supplementary file 1 [file nutrients-12-03420-s001.pdf]

Table S1. Dietary fiber intake in ALS participants according to tertiles of total fiber intake[1]

Variables	Total (*n* = 272)	Tertiles of total fiber intake (g)			*p*-value [2]
		T1 (*n* = 90) ≤13.45	T2 (*n* = 91) >13.45 to ≤19.84	T3 (*n* = 91) >19.84	
Vegetable fiber intake (g)	8.87 ± 6.08	4.23 ± 2.33 [3a]	7.95 ± 353 [b]	14.37 ± 6.37 [c]	<0.001
Fruit fiber intake (g)	2.75 ± 3.77	1.10 ± 1.56 [3a]	2.53 ± 2.53 [b]	4.60 ± 5.26 [c]	<0.001
Grain fiber intake (g)	2.89 ± 2.71	2.51 ± 1.47	2.77 ± 1.61	3.40 ± 4.13	0.456
Legume fiber intake (g)	2.42 ± 3.11	1.19 ± 1.49 [3a]	2.27 ± 2.62 [b]	3.79 ± 4.08 [c]	<0.001
Nut/seed fiber intake (g)	1.14 ± 1.17	0.60 ± 0.69 [3a]	0.99 ± 0.75 [b]	1.83 ± 1.51 [c]	<0.001

[1] Values are presented as means ± SD; [2] *p*-values were determined by ranked ANCOVA after adjustment for the age at symptom onset, sex, BMI, drinking habits, disease progression rate, and energy intake with Bonferroni's post hoc test; [3] Values with different superscript letters in the same row were significantly different at $p < 0.05$ according to ranked ANOVA with Bonferroni's post hoc test.

Table S2. Biochemical parameters of participants with ALS according to tertiles of total fiber intake[1]

Variables	Total (*n* = 272)	Tertiles of total fiber intake (g)			*p*-value [2]
		T1 (*n* = 90) ≤13.45	T2 (*n* = 91) >13.45 to ≤19.84	T3 (*n* = 91) >19.84	
FPG (mmol/L)	6.13 ± 1.53	6.12 ± 1.64	6.02 ± 1.39	6.24 ± 1.57	0.817
Total protein (g/L)	70.83 ± 5.33	70.45 ± 5.39	71.04 ± 5.12	71.00 ± 5.50	0.383
Albumin (g/L)	43.14 ± 3.29	42.94 ± 3.47	43.37 ± 3.16	43.09 ± 3.24	0.240
Creatinine (μmol/L)	62.18 ± 14.00	59.76 ± 14.94	64.32 ± 14.08	62.44 ± 12.68	0.273
BUN (mmol/L)	5.10 ± 1.38	5.30 ± 1.65	5.05 ± 1.22	4.94 ± 1.22	0.615
ALP (μkat/L)	1.03 ± 0.31	1.05 ± 0.34	1.05 ± 0.31	0.97 ± 0.26	0.455
AST (μkat/L)	0.44 ± 0.18	0.45 ± 0.22	0.44 ± 0.16	0.43 ± 0.15	0.475
ALT (μkat/L)	0.47 ± 0.30	0.46 ± 0.37	0.47 ± 0.26	0.49 ± 0.26	0.666
WBC (10^9/L)	6.78 ± 1.93	6.82 ± 2.14	6.66 ± 1.98	6.84 ± 1.67	0.565
Hemoglobin (g/L)	139.41 ± 14.20	138.89 ± 14.48	140.19 ± 14.63	139.14 ± 13.59	0.956
Hematocrit (%)	41.31 ± 3.90	41.13 ± 4.00	41.56 ± 4.04	41.25 ± 3.69	0.960
Lymphocyte (%)	31.41 ± 8.64	31.58 ± 8.96	30.47 ± 8.29	32.20 ± 8.68	0.546
TC (mmol/L)	4.85 ± 0.87	4.75 ± 0.76	4.94 ± 0.85	4.87 ± 0.97	0.401
TG (mmol/L)	1.61 ± 0.94	1.58 ± 0.84	1.66 ± 0.94	1.58 ± 1.01	0.465
HDL-C (mmol/L)	1.25 ± 0.30	1.24 ± 0.32	1.25 ± 0.29	1.26 ± 0.31	0.800
LDL-C (mmol/L)	2.86 ± 0.91	2.71 ± 0.72	3.03 ± 1.13	2.83 ± 0.80	0.282

FPG, fasting plasma glucose; BUN, blood urine nitrogen; ALP, alkaline phosphate; AST, aspartate aminotransferase; ALT, alanine aminotransferase; WBC, white blood cell; TC, total cholesterol; TG, triglyceride; HDL-C, high-density lipoprotein-cholesterol; LDL-C, low-density lipoprotein-cholesterol; [1] Values are presented as means ± SD; [2] *p*-values were determined by ranked ANCOVA after adjustment for the age at symptom onset, sex, BMI, drinking habits, disease progression rate, and energy intake.

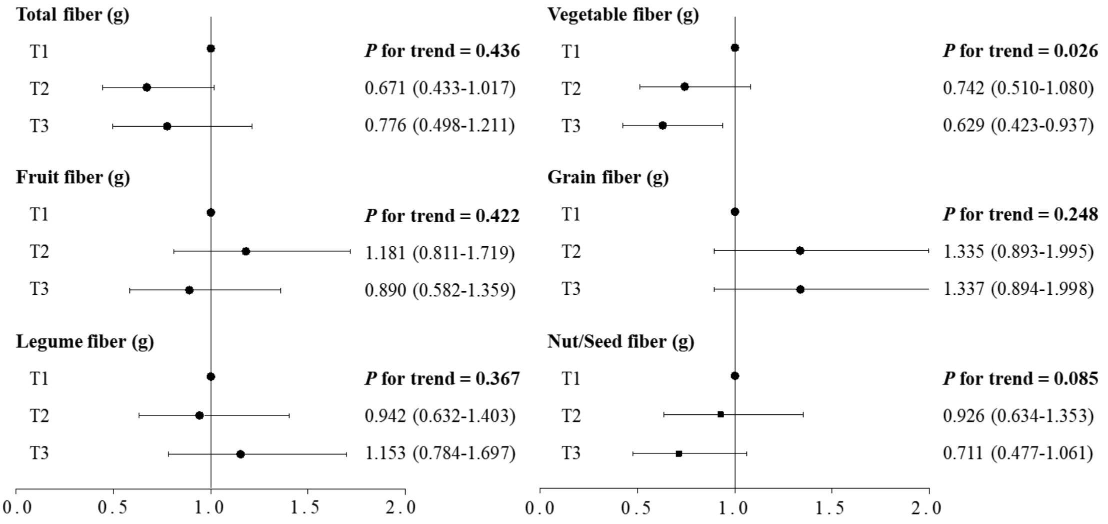

Figure S1. Cox proportional hazards regression analysis of event status in ALS participants according to tertiles of fiber intake. Adjusted HRs and 95% CIs were determined by cox proportional hazards regression analysis after adjustment for the age at symptom onset, sex, BMI, drinking habits, disease progression rate, and energy intake. HR, hazard ratio; Cl, confidence interval.
